# A multi-omic analysis of an Enterococcus faecium mutant reveals specific genetic mutations and dramatic changes in mRNA and protein expression

**DOI:** 10.1186/1471-2180-13-304

**Published:** 2013-12-28

**Authors:** De Chang, Yuanfang Zhu, Li An, Jinwen Liu, Longxiang Su, Yinghua Guo, Zhenhong Chen, Yajuan Wang, Li Wang, Junfeng Wang, Tianzhi Li, Xiangqun Fang, Chengxiang Fang, Ruifu Yang, Changting Liu

**Affiliations:** 1 Nanlou Respiratory Diseases Department, Chinese PLA General Hospital, Beijing 100853, China; 2 BGI-Shenzhen, Shenzhen, People’s Republic of China; 3 State Key Laboratory of Pathogen and Biosecurity, Beijing Institute of Microbiology and Epidemiology, Beijing 100071, China

**Keywords:** *E. faecium*, Genome, Transcriptome, Proteome, Multi-omics

## Abstract

**Background:**

For a long time, *Enterococcus faecium* was considered a harmless commensal of the mammalian gastrointestinal (GI) tract and was used as a probiotic in fermented foods. In recent decades, *E. faecium* has been recognised as an opportunistic pathogen that causes diseases such as neonatal meningitis, urinary tract infections, bacteremia, bacterial endocarditis and diverticulitis. *E. faecium* could be taken into space with astronauts and exposed to the space environment. Thus, it is necessary to observe the phenotypic and molecular changes of *E. faecium* after spaceflight.

**Results:**

An *E. faecium* mutant with biochemical features that are different from those of the wild-type strain was obtained from subculture after flight on the SHENZHOU-8 spacecraft. To understand the underlying mechanism causing these changes, the whole genomes of both the mutant and the WT strains were sequenced using Illumina technology. The genomic comparison revealed that *dprA*, a recombination-mediator gene, and *arpU*, a gene associated with cell wall growth, were mutated. Comparative transcriptomic and proteomic analyses showed that differentially expressed genes or proteins were involved with replication, recombination, repair, cell wall biogenesis, glycometabolism, lipid metabolism, amino acid metabolism, predicted general function and energy production/conversion.

**Conclusion:**

This study analysed the comprehensive genomic, transcriptomic and proteomic changes of an *E. faecium* mutant from subcultures that were loaded on the SHENZHOU-8 spacecraft. The implications of these gene mutations and expression changes and their underlying mechanisms should be investigated in the future. We hope that the current exploration of multiple “-omics” analyses of this *E. faecium* mutant will provide clues for future studies on this opportunistic pathogen.

## Background

In the past, *E. faecium* was considered to be a harmless commensal of the mammalian GI tract and was used as a probiotic in fermented foods [1,2]. In recent decades, *E. faecium* has been recognised as an opportunistic pathogen that causes diseases such as neonatal meningitis, urinary tract infections, bacteremia, bacterial endocarditis and diverticulitis [3-7]. Therefore, *E. faecium* can penetrate and survive in many environments in the human body, which could potentially lead to unpredictable consequences.

Due to revolutionary advances in high-throughput DNA sequencing technologies [8] and computer-based genetic analyses, genome decoding and transcriptome sequencing (RNA-seq) [9,10] analyses are rapid and available at low costs. Moreover, the development of mass spectrometry-based proteomic analysis provides a simple and convenient approach to identify and quantify thousands of proteins in a single experiment [11,12]. By employing these high-throughput technologies, the mechanisms underlying the systematic changes of a mutant and wild-type microbe could be revealed. Here we employed multi-omic technologies, including genomic, transcriptomic and proteomic analysis of a mutant strain of *E. faecium* and the corresponding wild-type strain to understand the complex mechanisms behind the mutations resulting in altered biochemical metabolic features.

## Methods

### Acquisition of the mutant

The *E. faecium* strain that was loaded in the SHENZHOU-8 spacecraft as a stab culture was obtained from the Chinese General Microbiological Culture Collection Center (CGMCC) as CGMCC 1.2136. After spaceflight from Nov. 1st to 17th, 2011, the *E. faecium* sample was struck out and grown on solid agar with nutrients. Then, 108 separate colonies were picked randomly and screened using the 96 GEN III MicroPlate^TM^ (Biolog, USA). The ground strain LCT-EF90 was used as the control. With the exception of spaceflight, all other culture conditions were identical between the two groups. The majority of selected subcultures showed no differences in the biochemical assays except for strain LCT-EF258. Compared with the control strain, a variety of the biochemical features of LCT-EF258 had changed after a 17-day flight in space. Based on the Biolog colour changes, strain LCT-EF258 had differences in utilisation patterns of N-acetyl-D-galactosamine, L-rhamnose, myo-inositol, L-serine, L-galactonic acid, D-gluconic acid, glucuronamide, p-hydroxy- phenylacetic acid, D-lactic acid, citric acid, L-malic acid and γ-amino-butryric acid relative to the control strain LCT-EF90 (Table 1). Despite isolation of this mutant, we could not determine if the underlying mutations were caused by the spaceflight environment. However, the mutant’s tremendous metabolic pattern changes still drew our interest to uncover possible genomic, transcriptomic and proteomic differences and to further understand the mechanisms underlying these differences.

**Table 1:** Phenotypic characteristics of the mutant (LCT-EF258) and the control strain (LCT-EF90) used in this study

**Features**	**LCT-EF90**	**LCT-EF258**
N-acetyl-D-galactosamine	-	+/−
L-rhamnose	-	+/−
Myo-inositol	-	+/−
L-serine	+/−	-
L-galactonic	-	+/−
D-gluconic acid	+/−	-
Glucuronamide	+/−	-
p-hydroxy- phenylacetic acid	+	-
D-lactic acid	-	+/−
Citric acid	+/−	-
L-malic acid	-	+
γ-amino-butryric acid	-	+

Note: “ + ” represents a significantly positive reaction; “+/−” represents a slightly positive reaction; “-” represents a negative reaction.

### DNA, RNA and protein preparation

Both the mutant and the control strains were grown in Luria-Bertani (LB) medium at 37°C; genomic DNA was prepared by conventional phenol-chloroform extraction methods; RNAs were exacted using TIANGEN RNAprep pure Kit (Beijing, China) according to the manufacturer’s instructions. Protein was extracted and quantified and was subsequently analysed by SDS-polyacrylamide gel electrophoretogram. After digestion with trypsin, the samples were labelled using the iTRAQ reagents (Applied Biosystems), which fractionates the proteins using strong cationic exchange (SCX) chromatography (Shimadzu). Each fraction was separated using a splitless nanoACQuity (Waters) system coupled to the Triple TOF 5600 System (AB SCIEX, Concord, ON).

### Genome sequencing and annotation

#### Sequencing and filtering

Using genomic DNA from the two samples, we constructed short (500 bp) and large (6 kb) random sequencing libraries and selected 90-bp read lengths for both libraries. Raw data were generated from the Illumina Hiseq2000 next-generation sequencing (NGS) platform with Illumina 1.5 format encoding a Phred quality score from 2 to 62 using ASCII 66 to 126. The raw data were then filtered through four steps, including removing reads with 5 bp of Ns’ base numbers, removing reads with 20 bp of low quality (≤Q20) base numbers, removing adapter contamination, and removing duplication reads. Finally, a total of 55 million base pairs of reads were generated to reach a depth of ~190-fold of total genome coverage.

#### Repetitive sequences analysis

We searched the genome for tandem repeats using Tandem Repeats Finder [13] and Repbase [14] (composed of many transposable elements) to identify the interspersed repeats. Transposable elements in the genome assembly were identified both at the DNA and protein level. For identification of transposable elements at the DNA level, RepeatMasker [15] was applied using a custom library comprising a combination of Repbase. At the protein level, RepeatProteinMask, which is updated software in the RepeatMasker package, was used to perform RM-BlastX against the transposable elements protein database.

#### ncRNA sequences analysis

The tRNA genes were predicted by tRNAscan [16]. Aligning the rRNA template sequences from animals using BlastN with an E-value of 1e-5 identified the rRNA fragments. The miRNA and snRNA genes were predicted by INFERNAL software [17] against the Rfam database [18].

#### Gene functional annotation

To ensure the biological meaning, we chose the highest quality alignment result to annotate the genes. We used BLAST to accomplish functional annotation in combination with different databases. We provided BLAST results in m8 format and produced the annotation results by alignment with selected databases.

#### Nucleotide sequence accession number

The whole-genome sequences of the wild-type and mutant *E. faecium* strains in this study have been deposited at DDBJ/EMBL/GenBank under the accession numbers ANAJ00000000 and ANAI00000000, respectively.

### Comparative genomic analysis

#### SNPs calling

Raw SNPs were identified using software MUMmer (Version 3.22) [19] and SOAPaligner (Version 2.21). In all, raw SNPs were filtered by the following criteria: SNPs with quality scores < 20, SNPs covered by < 10 paired-end reads, SNPs within 5 bp on the edge of reads, and SNPs within 5 bp of two or more existing mutations. Finally, SNPs in repetitive regions found using the “Repetitive sequences analysis” method were also filtered.

#### Small size InDel variants calling

First, InDels (insertions and deletions) with lengths of less than 10 bp were extracted from the gap extension alignment between the genome assembly and the reference using LASTZ (Version 1.01.50). Second, we removed the unreliable InDels containing N base within 50 bp upstream and downstream, and we removed InDels with more than two mismatches within a total of 20 bp upstream and downstream. Finally, the candidate InDels were verified by comparing sample reads to the surrounding region of the InDels (100 bp each side) with the reference sequence by using BWA (Version 0.5.8) [20].

#### Synteny analysis

The LCT-EF258 target sequences were ordered according to the reference sequence based on MUMmer. Then, the X and Y axes of the two-dimensional synteny graphs and the upper and following axes of linear syntenic graphs were constructed after the same proportion of size reduction in the length of both sequences. The protein set P1 of the target sequence was aligned with the protein set P2 of the reference sequence using BLASTP (e-value < = 1e-5, identity > = 85%, and the best hit of each protein was selected). Finally, the results with the best-hit value were reserved and the average of two consistent values was obtained.

### Transcriptome sequencing and comparison

#### Sequencing and filtering

Total RNAs were purified using TRIzol (Invitrogen) and rRNA was removed. Then, cDNA synthesis was performed with random hexamers and Superscript II reverse transcriptase (Invitrogen). Meanwhile, double-stranded cDNAs were purified with a Qiaquick PCR purification kit (Qiagen) and sheared with a nebuliser (Invitrogen) to ~200 bp fragments. After end repair and poly (A) addition, the cDNAs were ligated to Illumina N-acetyl-D-galactosamine (pair end) adapter oligo mix and suitable fragments were selected as templates by gel purification. Next, the libraries were PCR amplified and were sequenced using the Illumina Hiseq 2000 platform and the paired-end sequencing module.

The filtration consisted of three steps: removing reads with 1 bp of Ns’ base numbers, removing reads with 40 bp of low quality (≤Q20) base numbers, and removing adapter contamination. Additionally, reads mapped to the reference (LCT-EF90) rRNA sequences were removed. All gene expression data generated in this study have been deposited under accession numbers SRR922447 and SRR922448 (https://trace.ddbj.nig.ac.jp/DRASearch/).

#### Gene expression value statistics

The gene coverage was evaluated by mapping clean reads to the reference genes using SOAPaligner software, and the gene expression value was calculated by the RPKM (Reads Per kb per Million reads) formula based on the method described in Ali et al. [21]. The RPKM method was able to eliminate the influence of gene length and sequencing discrepancy on the gene expression calculation. Therefore, the calculated gene expression could be directly used for comparing the gene expression among difference samples.

#### Differential gene expression analysis

To control error rate and identify true differentially expressed genes (DEGs), the p-value was rectified using the FDR (False Discovery Rate) control method [22]. Both the FDR value and the RPKM ratio in different samples were calculated. Finally, genes with an RPKM ratio ≥ 2 and a FDR ≤ 0.001 between different samples were defined as DEGs. Different DEGs were enriched and clustered according to the GO and KEGG functions.

### Proteomic study

Quantitative proteomics were performed using iTRAQ technology coupled with 2D-nanoLC-nano-ESI-MS/MS to examine the difference of protein profiles [23]. After identification by the TripleTOF 5600 System, data acquisition was performed with a TripleTOF 5600 System (AB SCIEX, Concord, ON) fitted with a Nanospray III source (AB SCIEX, Concord, ON) with a pulled quartz tip as the emitter (New Objectives, Woburn, MA). Data analysis, including protein identification and relative quantification, were performed with the ProteinPilotTM software 4.0.8085 using the Paragon Algorithm version 4.0.0.0 as the search engine. Each MS/MS spectrum was searched against the genome annotation database (5263 protein sequences), and the search parameters allowed for Cys. The local FDR was set to 5%, and all identified proteins were grouped by the ProGroup algorithm (ABI) to minimise redundancy. Proteins were identified based on at least one peptide with a percent confidence above 95%. Some of the identified peptides were excluded according to the following conditions: (i) Peptides with low ID confidence (<15%) were excluded. (ii) Peptide peaks corresponding to the ITRAQ labels were not observed. (iii) Shared MS/MS spectra, due to either identical peptide sequences in more than one protein or when more than one peptide was fragmented simultaneously, were excluded. (iv) Any peptide ratio in which the S/N (signal-to-noise ratio) is too low was excluded. Several quantitative estimates provided for each protein by the Protein Pilot were utilised, including the fold change ratios of differential expression between labelled protein extracts and the P value, which represents the probability that the observed ratio is different to 1 by chance. All experiments were performed in three replicates, and the differentially expression proteins (DEPs) were selected if they appeared at least twice and the fold change was larger than 1.2 with a p-value less than 0.05. The mass spectrometry proteomics data have been deposited to the ProteomeXchange Consortium (http://proteomecentral.proteomexchange.org) via the PRIDE partner repository with the dataset identifier PXD000326.

### Bioinformatics analysis

#### Gene ontology and GO enrichment analysis

GO (Gene Ontology) enrichment analysis provided all GO terms that were significantly enriched in a list of DEGs, and the DEGs were filtered corresponding to specific biological functions. We first mapped all DEGs to GO terms in the database, calculating gene numbers for every term, and then used the hypergeometric test to find significantly enriched GO terms based on GO::TermFinder [24]. Here, a strict algorithm was developed for the analysis:

$$P=1‒\sum_{i=0}^{m-1}\frac{\left({}_{i}^{M}\right)\left({}_{n-i}^{N-M}\right)}{{}_{n}^{N}}$$

where N was the number of all genes with GO annotation; n was the number of DEGs in N; M was the number of all genes that were annotated to certain GO terms; m was the number of DEGs in M. The calculated p-value required a corrected p-value ≤ 0.05 as a threshold by Bonferroni correction.

#### Pathway analysis and pathway enrichment analysis

Gene interactions play key roles in many biological functions. Pathway enrichment of DEGs was analysed by the KEGG pathway [25]. This analysis identified significantly enriched metabolic pathways in DEGs when compared with the genome background. The same analysis utilized in the GO enrichment was used for the pathway enrichment analysis. Here, N was the number of all genes with KEGG annotation, n was the number of DEGs in N, M was the number of all genes annotated to specific pathways, and m was the number of DEGs in M.

#### COG function analysis

Cluster of Orthologous Groups of proteins (COG) is the database for gene/protein orthologous classification (http://www.ncbi.nlm.nih.gov/COG/). Every gene/protein in a COG is supposed to be derived from a single gene/protein ancestor. Orthologs are gene/proteins derived from different species of one vertical family and have the same functions as the ancestor. Paralogs are proteins derived from gene expression and may have new, related functions. We compared identified proteins with the COG database to predict the gene or proteins’ function.

## Results

### Genomic sequencing, assembly and annotation

Genomic DNA from both samples was sequenced using a whole-genome shotgun sequencing (WGS) approach on the Illumina Hiseq2000 system. The short (500 bp) and large (6 kb) random sequencing libraries were constructed, and the mean read length was 90 bp for both libraries. A total of 55 million base pairs of reads were generated to reach a depth of ~190-fold genome coverage (see Methods for details). The genomes were assembled using SOAPdenovo (Version 1.05) [26], which resulted in the final high quality genomic assemblies.

**Figure 1:**
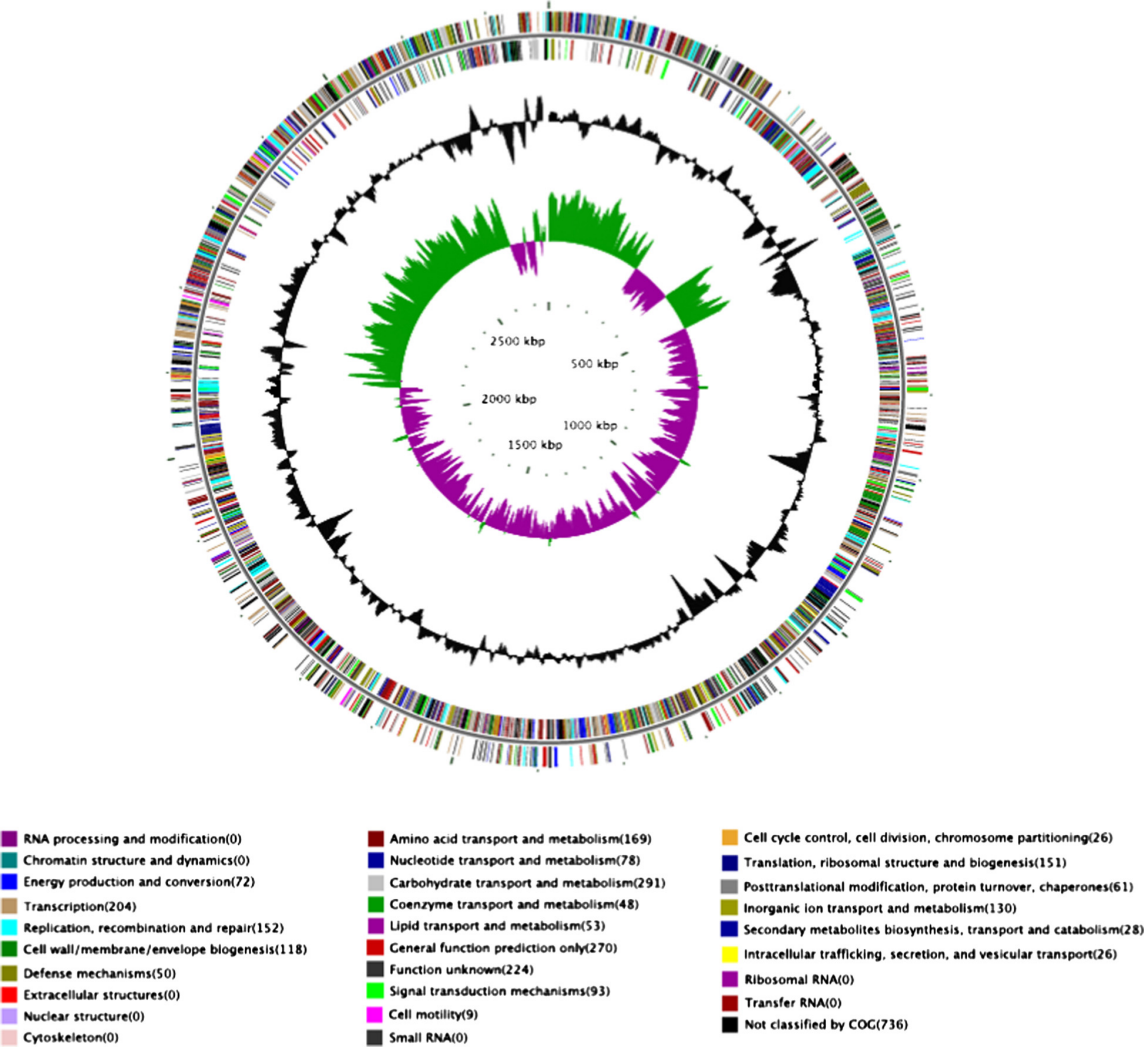
**Genome map of E. faecium strain LCT-EF90 (ncRNA, COG annotation, GC content and GC skew).** From outer to innner, the 1st circle shows the ncRNA result of the positive strand containing tRNA, rRNA and sRNA; the 2nd circle showed the COG function of the positive strand along scaffolds and each colour represents a function classification; the 3rd circle shows the ncRNA result of negative strand; the 4th circle shows the COG function of the negative strand; the 5th circle shows the GC content (black); the 6th circle shows the GC skew ((G-C)/(G + C), green > 0, purple < 0). The 5th and 6th circle are plotted in relation to the average value.

Before the comparative genomics analysis, gene models and their associated functions for strain LCT-EF90 were determined using different databases. First, we used Glimmer software [27] for gene prediction and identified 2,777 genes with a total length of 2,394,186 bp, which consisted of 86.31% of the genome. In addition, 13,090 bp of the transposon sequences and 4,787 bp of the tandem repeat sequences were identified, which consisted of 0.47% and 0.17% of genome, respectively (Additional file 1: Table S1). We identified 37 tRNA fragments with a total length of 2,807 bp and 2 snRNA (small nuclear RNA) genes with a total length of 367 bp (see Methods for details). We annotated all of the genes against the popular functional databases, including 59.60% of the genes into the GO database (Additional file 1: Figure S1) [28], 73.50% of the genes into COG (Additional file 1: Figure S2) [29], 66.69% of the genes into KEGG (Additional file 1: Figure S3) [25], 97.34% of the genes into the NR database, 69.07% genes into SwissProt [30] and 97.34% of the genes into TrEMBL [31] (see Methods for details). Moreover, 321 genes were identified in the CAZY (Carbohydrate-Active enzymes) database [32], 210 genes in the PHI-base (Pathogen - Host Interaction) database [33], 6 genes in DBETH (a Database of Bacterial Exotoxins for Human) [34] and 387 genes in VFDB (Virulence Factors Database) [35]. In addition, our analysis predicted genome islands, prophages and CRISPRs (Clustered Regularly Interspaced Short Palindromic Repeats), but no CRISPRs have been found. The genome map of E. faecium strain LCT-EF90 was shown in Figure 1.

### Comparative genomic analysis

**Figure 2:**
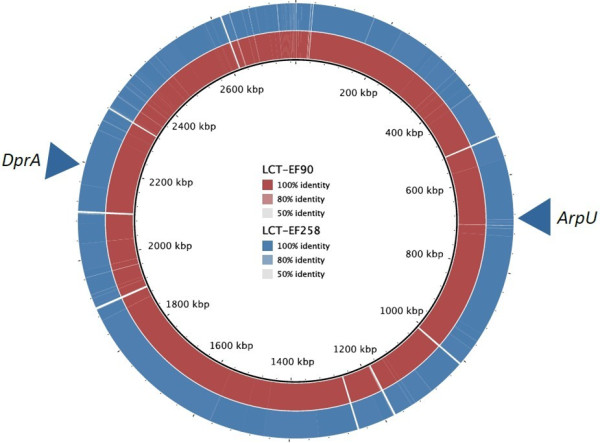
**Comparative genomic analysis.** We used BRIG software to achieve alignment results of three genomes. The gray circle is LCT-EF90, and blue circle is LCT-EF258. There are some white regions in two circles, which are the gaps in genomes. The triangles indicate the general positions of the mutations with SNPs and InDels, which were annotated into genes *dprA* and *arpU*.

We used LCT-EF90 as the reference strain and detected variations, including SNPs, InDels and structure variations (SVs) between LCT-EF258 and LCT-EF90 (Figure 2). For SNP identification, the query sequence was aligned with the reference sequence using MUMmer software (Version 3.22) [36] (see Methods for details). The raw variation sites were identified and then filtered with strict standards to detect potential SNP sites. Finally, 1 SNP for *E. faecium* LCT-EF258 was detected and was located in the functional gene LCT-EF90GL001983 (Additional file 1: Table S2). The SNP mutation in LCT-EF90GL001983 was a non-synonymous substitution in *dprA*, a gene encoding a DNA processing protein based on KEGG pathway analysis, and may play an important role in phenotypic variation.

To detect more variations, we used the LASTZ (Version 1.01.50) tool to identify InDels less than or equal to 10 bp (see Methods for details). After a series of filtering conditions, we have found 8 InDels between LCT-EF90 and LCT-EF258 (Additional file 1: Table S3), including 7 InDels in intergenic regions and only one in a coding region. The coding region InDel was identified in LCT-EF90GL000008, which is annotated as an *arpU* family gene related to transcriptional regulators in the NR database (Additional file 1: Table S4) but not in VFDB (Virulence Factors Database). While small size InDels were found in sample LCT-EF258, we were also interested in large scale structural variations. We aligned the two samples with a reference at the nucleic acid level (see Methods for details) but did not identify any large scale SVs. The probable reason may be that the generation time was so short that the variations did not have enough time to accumulate.

### Transcriptomic analysis

**Figure 3:**
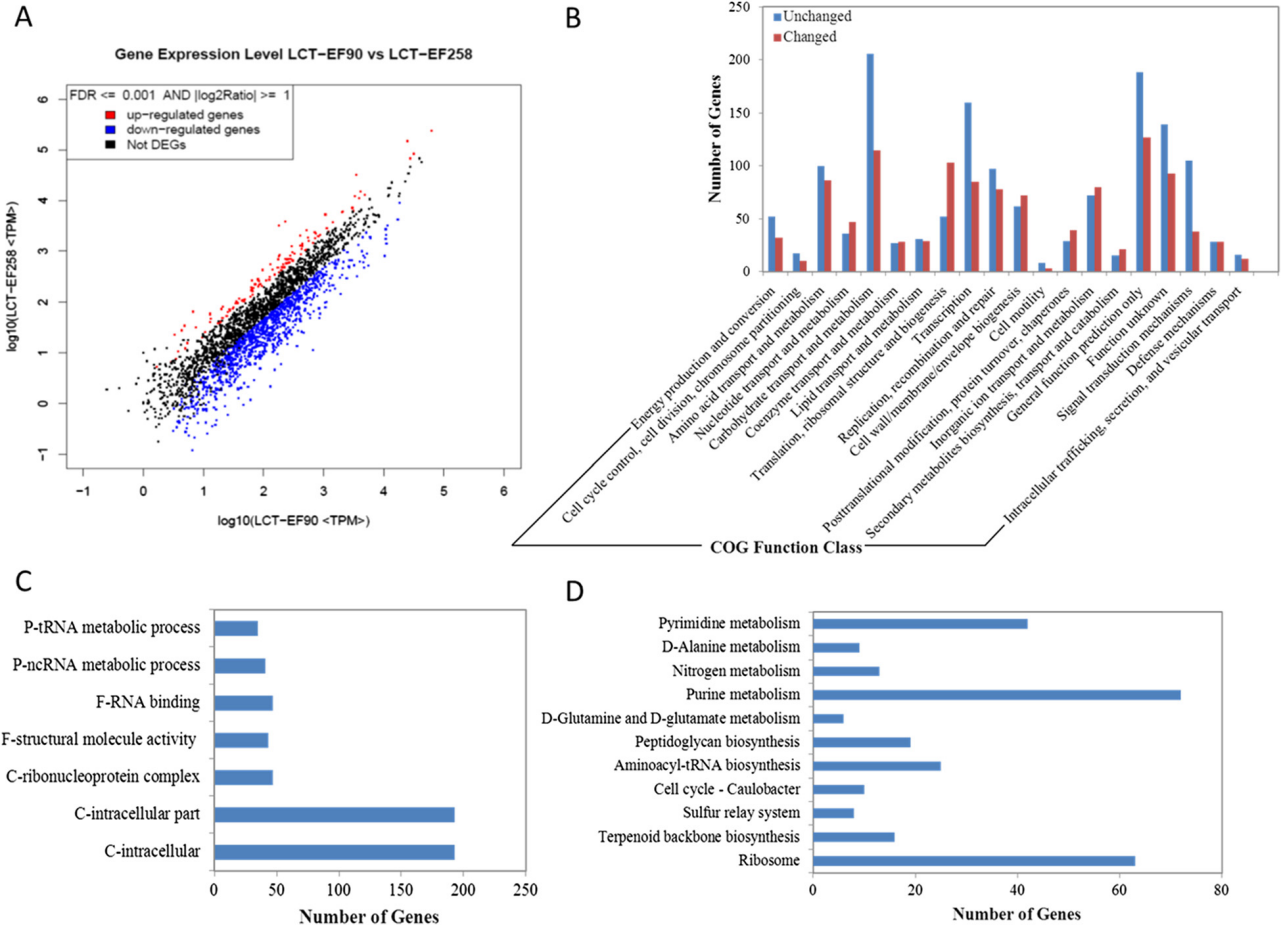
**Differential transcriptomic analysis. (A)**. Global profiling of gene expression changes. Here |log2Ratio| was the log2ratio of LCT_EF258/LCT_EF90, and TPM was defined by tags per million. **(B)**. Clustered DEGs in COG between LCT-EF90 and LCT-EF258. **(C)**. Clustered DEGs in GO between LCT-EF90 and LCT-EF258. The x-axis represents the number of the genes corresponding to the GO functions. The y-axis represents GO functions. **(D)**. Clustered DEGs in KEGG between LCT-EF90 and LCT-EF258. The x-axis represents the number of the genes corresponding to the KEGG pathways. The y-axis represents KEGG pathways.

Using gene difference expression analysis, 2,679 genes between LCT-EF90 and LCT-EF258 were detected. After filtering conditions of FDR ≤ 0.001 and RPKM Ratio ≥ 2, 1,159 genes remained. Both up-regulated and down-regulated genes were identified in this analysis. Approximately 123 genes were up-regulated, and 1,036 genes were down-regulated between LCT-EF90 and LCT-EF258 (Figure 3A). We found that the down-regulated genes significantly out-numbered up-regulated genes, suggesting that gene expression and metabolism were inhibited in LCT-EF258.

Different DEGs were enriched and clustered according to GO, COG and KEGG analyses. For COG, the up-regulated and down-regulated genes were summed and were compared with unchanged genes. The most change was annotated into the translation, ribosomal structure and biogenesis function classes (Figure 3B). For gene ontology, the DEGs that showed statistical significance (P-value ≤0.05) were the component, function and process ontologies. For LCT-EF90 and LCT-EF258, seven categories, including 601 DEGs (identical DEGs may fall into different categories), were shown to be meaningful (Figure 3C). For the KEGG functional cluster, there were eleven categories, including 283 DEGs, between LCT-EF90 and LCT-EF258. Most of the genes were annotated into three categories: purine metabolism, pyrimidine metabolism and ribosome (Figure 3D).

### Comparative proteomic analysis

**Figure 4:**
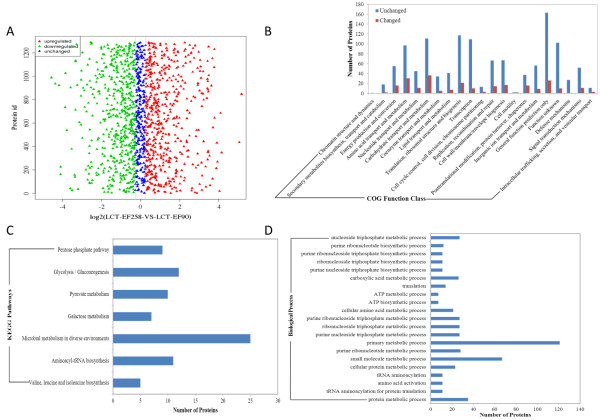
**Comparative proteomic analysis. (A)**. Protein ratio distribution. The distribution of average value of protein quantification in three repeated experiments is shown. Red: fold change > 1.2, Green: fold change < −1.2. **(B)**. COG function analysis of differentially expressed proteins. **(C)**. KEGG pathways analysis of proteins with different expression (P value <0.05). **(D)**. Gene ontology enrichment analysis of differentially expressed proteins. GO terms of biological process were analysed and significantly enriched catalogues are shown (P-value < 0.01).

Using Protein Pilot software, 1188 proteins that appeared at least twice in three replicates were identified [37]. Relatively quantitative analysis shows that 213 DEPs were identified, including 116 down-regulated proteins and 97 up-regulated proteins (Figure 4A). Subsequently, DEPs were classified according to COG function category. It is clear that the expression of proteins involved in functions such as energy production, metabolism, transcription, translation, posttranslational modification, DNA recombination and repair, cell wall biogenesis and signal transduction mechanisms changed the most (Figure 4B). The enrichment and cluster of DEPs were performed according to Gene Ontology and KEGG Pathways functional analysis. The metabolic and biosynthetic biological processes were found to be different in the mutant (Figure 4C). As to KEGG functions affected in the mutant, significant difference was found in the following pathways: valine, leucine and isoleucine biosynthesis; aminoacyl-tRNA biosynthesis; pyruvate metabolism; galactose metabolism; glycolysis; pentose phosphate pathway; and microbial metabolism in diverse environments (Figure 4D).

### Integration of transcriptomic and proteomic analysis

**Figure 5:**
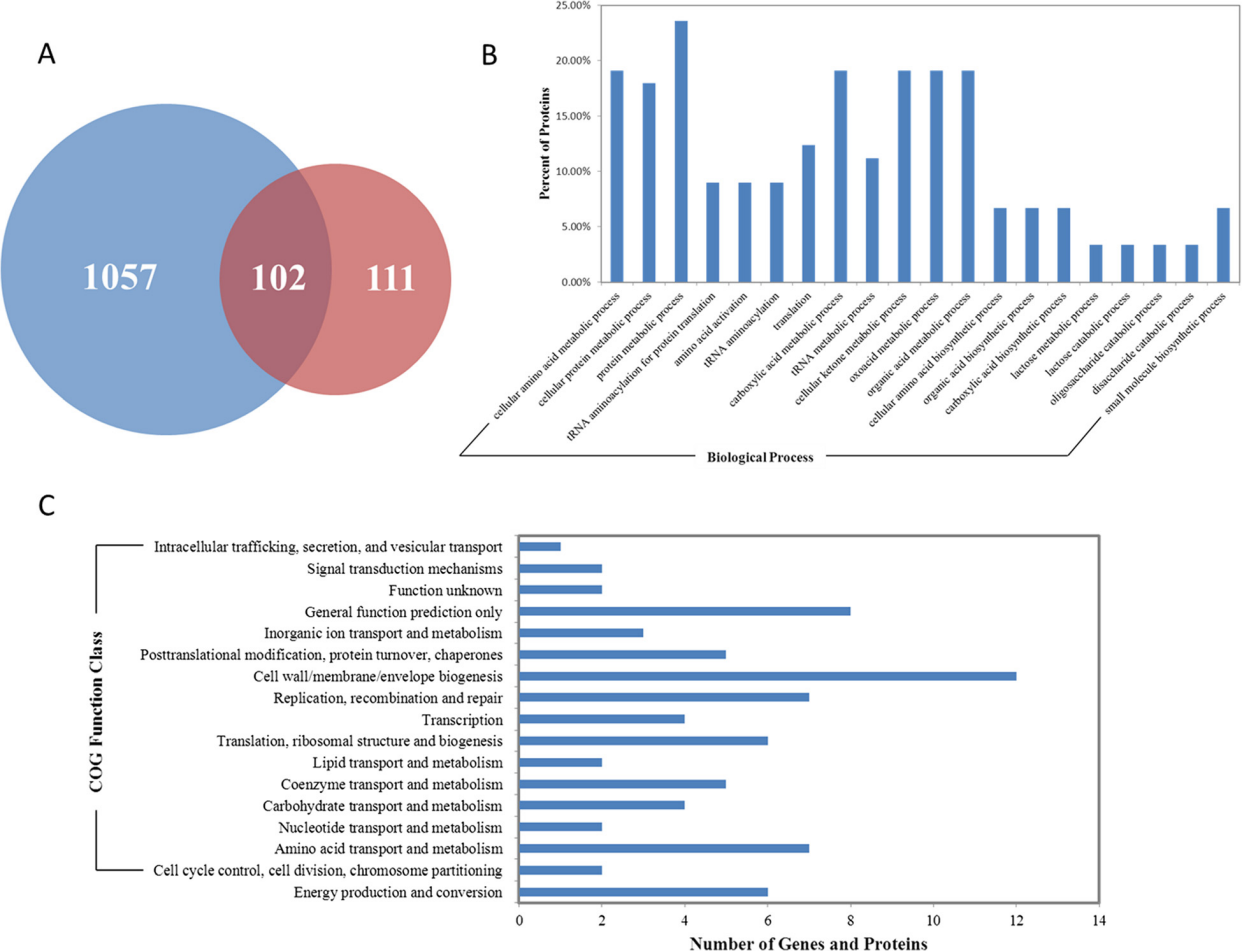
**Integration of the transciptome and the proteome. (A)**. The overlaps of DEGs and DEPs were analysed (The DEGs were genes with RPKM ratios ≥ 2 and a FDR ≤ 0.001; the DEPs were proteins that appeared at least twice in three replicates). **(B)**. GO enrichment analysis of overlaps between DEGs and DEPs. GO terms of biological process were analysed and significantly enriched catalogues are shown (P-value < 0.01). **(C)**. Clustered DEGs in COG function analysis of overlaps between DEGs and DEPs.

Most previous studies suggest a weak correlation between mRNA expression and protein expression, which may be due to post-transcriptional regulation of protein synthesis, post-translational modification or experimental errors [38-40]. However, according to the central dogma of molecular genetics, genetic information is transmitted from DNA to message RNAs that are subsequently translated to proteins [41,42]. Thus, we integrated the DEFs and DEPs to identify the overlapping genes that are expressed differently in both the transcriptome and the proteome. One-hundred and two genes were selected (Figure 5A), and those genes with either up-regulated or down-regulated expression at both the mRNA and protein levels were subjected to bioinformatic analysis. The Gene Ontology study indicated that biological processes such as metabolic processes, catabolic processes, biosynthetic processes and translation may be affected in the mutant strain (Figure 5B). Functional classification according to COG function category indicates that, except for the general function prediction catalogue and the amino acid transport and metabolism catalogue, the genes with the greatest change in expression are classified into the cell wall/membrane/envelope biogenesis and replication catalogue and the recombination and repair catalogue (Figure 5C). Interestingly, the genetic comparison revealed that gene mutations were identified in *dprA* and *arpU*. The former gene was described as a competence gene involved in the protection of incoming DNA, and the latter gene was a transcriptional regulator that plays a role in cell wall growth and division [43].

## Discussion

**Figure 6:**
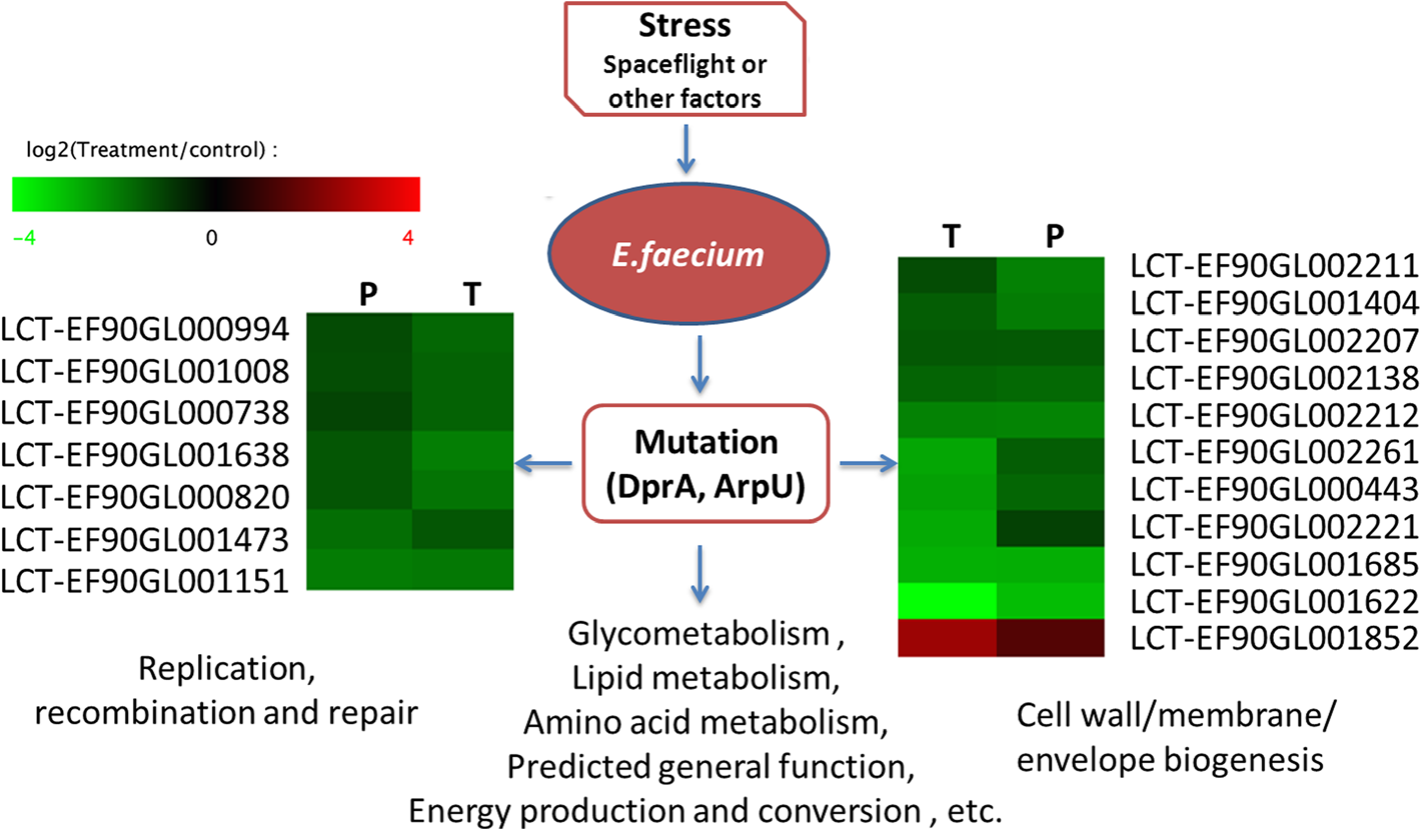
**Schematic representation of possible multi-omic alternations of E. faecium mutant.** The *dprA* and *arpU* mutations were the homozygous mutations identified in the gene-coding region, which may result in the transcriptomic and proteomic level changes of genes clustered into replication, recombination, repair, cell wall biogenesis, metabolisms, energy production and conversion and some predicted general function. “P” represents proteomic changes and “T” represents transcriptomic changes.

*E. faecium* is a part of the normal flora in human and animal intestines and is a ubiquitous opportunistic nosocomial pathogen. *E. faecium* was isolated from spacecraft-associated environments for the first time in 2009 [44]. Immune system suppression may make crew members susceptible to *E. faecium* during spaceflight*.* Furthermore, the virulence of *E. faecium* may be enhanced during spaceflight. There is no comprehensive genetic information currently available for *E. faecium* after spaceflight, which makes it difficult to study the pathogenicity of the organism after exposure to this unique environment. We originally planned to research the impact of spaceflight environments on bacteria using *E. faecium* as a model. However, because the subculture may also produce unknown mutations, we cannot exclusively determine that the mutations identified after spaceflight were caused by the spaceflight environment. However, we did not obtain any mutants from the ground control strain subcultures. We were still interested in revealing the possible mechanisms of the mutant compared to the control strain using multiple ‘omics’ analysis. This study presents the whole genome, transcriptome and proteome of a mutant *E. faecium* strain. Our results show that 2,777 genes were predicted, and two point mutations were identified and were located in *dprA* and a transcriptional regulator (ArpU family). DprA was described as a member of a recombination-mediator protein family, which is required for natural transformation relating to horizontal gene transfer in bacteria [45-48]. ArpU was reported to control the muramidase-2 export, which plays an important role in cell wall growth and division. Mutation of *arpU* may lead to serious metabolic effects [43]. The transcriptome and proteome analysis suggests that the differentially expressed genes and proteins are mainly distributed in pathways involved in glycometabolism, lipid metabolism, amino acid metabolism, predicted general function, energy production and conversion, replication, recombination and repair, cell wall, membrane biogenesis, etc. Among these changes, the two main altered functional classifications were the replication, recombination and repair catalogue and the cell wall and membrane biogenesis catalogue, which are in accordance with the predicted functions of the mutated genes. Expression changes of genes in the replication, recombination and repair catalogue may be caused by a stress-induced *dprA* mutation. The *arpU* mutation may affect the expression of members attributed to cell wall and membrane biogenesis (Figure 6). All of these changes at the molecular level may be caused by a stimulus during space flight. Because spacecraft are designed to provide an internal environment suitable for human life (reducing harmful conditions, such as high vacuum, extreme temperatures, orbital debris and intense solar radiation), *E. faecium* was placed in the cabin of the SHENZHOU-8 spacecraft to determine how microgravity as an external stimulus influences this bacterium.

## Conclusion

This study was the first to perform comprehensive genomic, transcriptomic and proteomic analysis of an *E. faecium* mutant*,* an opportunistic pathogen often present in the GI tract of space inhabitants. We identified *dprA* and *arpU* mutations, which affect genes and proteins with different expressions clustered into glycometabolism, lipid metabolism, amino acid metabolism, predicted general function, energy production, DNA recombination and cell wall biogenesis, etc. We hope that the current exploration of multiple “-omics” analyses of the *E. faecium* mutant could aid future studies of this opportunistic pathogen and determine the effects of the space environment on bacteria. However, the biochemical metabolism of bacteria is so complex that the biological meanings underlying the changes of *E. faecium* in this study is not fully understood. The implications of these gene mutations and expressions, and the mechanisms between the changes of biological features and the underlying molecular changes, should be investigated in the future. Moreover, the high cost of loading biological samples onto spacecraft and the difficult setting limits this type of exploration.

## Competing interests

The authors declare that there are no competing interests.

## Author’s contributions

All authors proposed and designed the study. DC performed the approach and analyzed the results. All authors contributed to the writing of the manuscript. All authors read and approved the final manuscript.

## Supplementary Material

Additional file 1: Tables S1, S2, S3, S4 Shows the repeat sequences statistics, SNP, Indels between LCT-EF258 and LCT-EF90, and annotation of InDels respectively. Supplementary figure represent function annotation in GO, GOG and KEGG database. Click here for file
