# Supplementary material for: A multi-omic analysis of an Enterococcus faecium mutant reveals specific genetic mutations and dramatic changes in mRNA and protein expression
Source: BMC Microbiol. 2013 Dec 28;13:304. doi: 10.1186/1471-2180-13-304 (PMC3879163; doi:10.1186/1471-2180-13-304)

**Supplement**

**Supplementary Tables**

**Table 1. Repeat sequences statistics**

| **Type** | **Repeat Size (bp)** | **In Genome (%)** |
| --- | --- | --- |
| **Repbase** | 8231 | 0.2967 |
| **ProMask** | 4859 | 0.1752 |
| **TRF** | 4787 | 0.1726 |
| **Total** | 17141 | 0.6179 |

**a.** Repbase represents transposon sequences identified by RepeatMasker software; ProMask represents the repeats identified by RepeatProteinMasker software; TRF represents tandem repeat sequences predicted using Tandem Repeat Finder.

Table 2. SNP results between sample LCT-EF258 and reference LCT-EF90

| **Position** | **Refer**  **Base** | **EF258**  **Base** | **Reference**  **Gene** | **Mutant**  **Type** | **Refer**  **amino** | **Mutant**  **amino** | **NR** | **KEGG** | **COG** |
| --- | --- | --- | --- | --- | --- | --- | --- | --- | --- |
| S3_  60853 | C | A | LCT-EF90  GL001983 | nonsyn | S | I | DNA protecting protein DprA | DNA processing protein DprA, K04096 DNA processing protein | Predicted Rossmann fold nucleotide-binding protein involved in DNA uptake |

**a.** Position indicates the LCT-EF90 scaffold position. **b.** LCT-EF90GL001983 was predicted by Glimmer software. **c.** nonsyn indicates a non-synonymous mutation. **d.** S is the abbreviation of [serine](app:ds:serine dehydrase) and I is the abbreviation of isoleucine.

Table 3. InDels annotation

| EF258 Scaff  Name | InDel Type | EF258 Start Pos | EF258 End Pos | Ref  Scaff  Name | Ref  Start Pos | Ref  End  Pos | InDel | Strain | InDel  annotation |
| --- | --- | --- | --- | --- | --- | --- | --- | --- | --- |
| S1 | Deletion | 9693 | 9693 | S1 | 5986 | 5987 | A | + | Intergenic |
| S1 | Insertion | 309588 | 309589 | S1 | 303354 | 303354 | A | + | Intergenic |
| S1 | Insertion | 309605 | 309611 | S1 | 303370 | 303370 | ACCTCT | + | Intergenic |
| S1 | Insertion | 319899 | 319909 | S1 | 313658 | 313658 | AATTTGGCTT | + | Intergenic |
| S1 | Insertion | 320100 | 320101 | S1 | 313849 | 313849 | T | + | Intergenic |
| S1 | Insertion | 320222 | 320223 | S1 | 313970 | 313970 | T | + | Intergenic |
| S1 | Insertion | 541285 | 541294 | S1 | 531950 | 531950 | CAGGTCAAA | + | Intergenic |
| S1 | Insertion | 706744 | 706746 | S1 | 696279 | 696279 | TC | + | LCT-EF90  GL000008 |

**a.** The first column is the LCT-EF258 scaffold name and the fifth column is the reference scaffold name. **b.** LCT-EF90GL000008 was predicted by Glimmer software.

Table 4. InDels between sample LCT-EF258 and reference LCT-EF90

| **Sample** | **InDel**  **Number** | **Insertion** | **Deletion** | **InGene**  **Number** | **InterGene**  **Number** | **Gene**  **Name** | **NR** | **KEGG** | **COG** |
| --- | --- | --- | --- | --- | --- | --- | --- | --- | --- |
| LCT-EF258 | 8 | 7 | 1 | 1 | 7 | LCT-EF90  GL000008 | transcriptional regulator,  ArpU family | NA | NA |

**a.** LCT-EF90GL000008 was predicted by Glimmer software. **b.** “NA” indicates that this gene has no functional annotation in KEGG and the COG database.

**Supplementary Figures**

**Figure 1. GO database function annotation.**

**a.** The x-axis represents three GO terms including cellular component, molecular function and biological process. **b.** The y-axis represents the number and percentage of the genes corresponding to the GO terms. **c.** The number of the genes annotated as possessing binding and catalytic activities are the most prevalent in the molecular function modules.

**Figure. 2. GOG database function annotation.**

1. The x-axis represents COG functional annotations. **b.** The y-axis represents the number of the genes corresponding to the COG functions. **c.** The carbohydrate transport and metabolism (G) COG contains the most genes.

**Figure. 3. KEGG database function annotation.**

**a.** The x-axis represents the number of the genes corresponding to the pathway. **b.** The y-axis represents KEGG pathways. **c.** The carbohydrate metabolism and membrane transport KEGG pathways contain the most genes.

**Supplementary Figure 1**


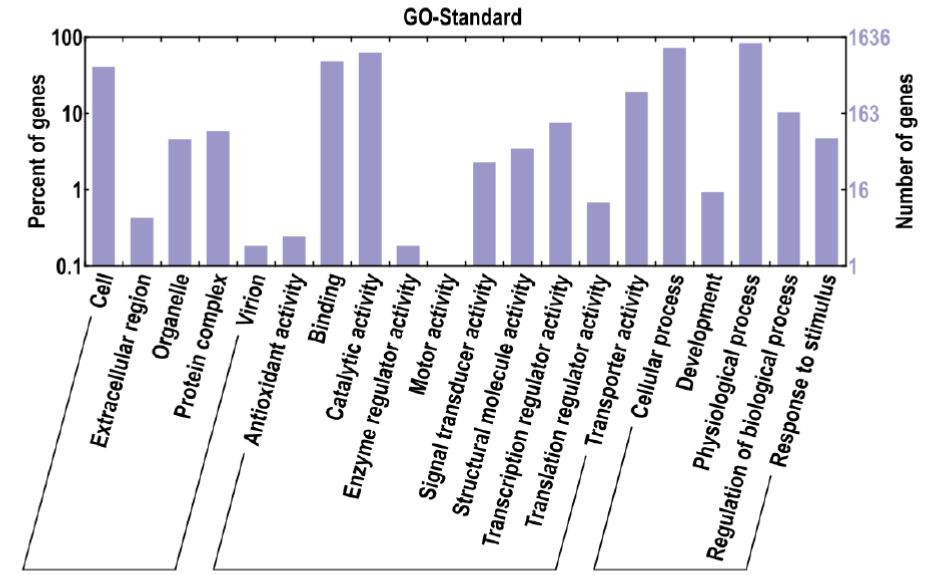


**Supplementary Figure 2**


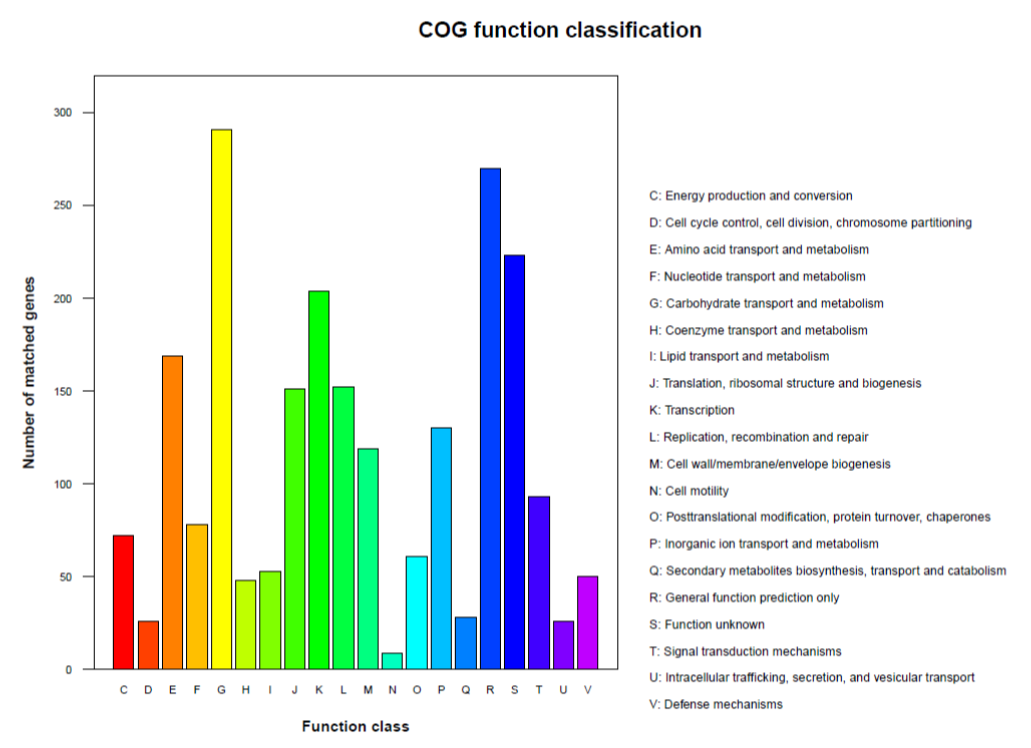


**Supplementary Figure 3**


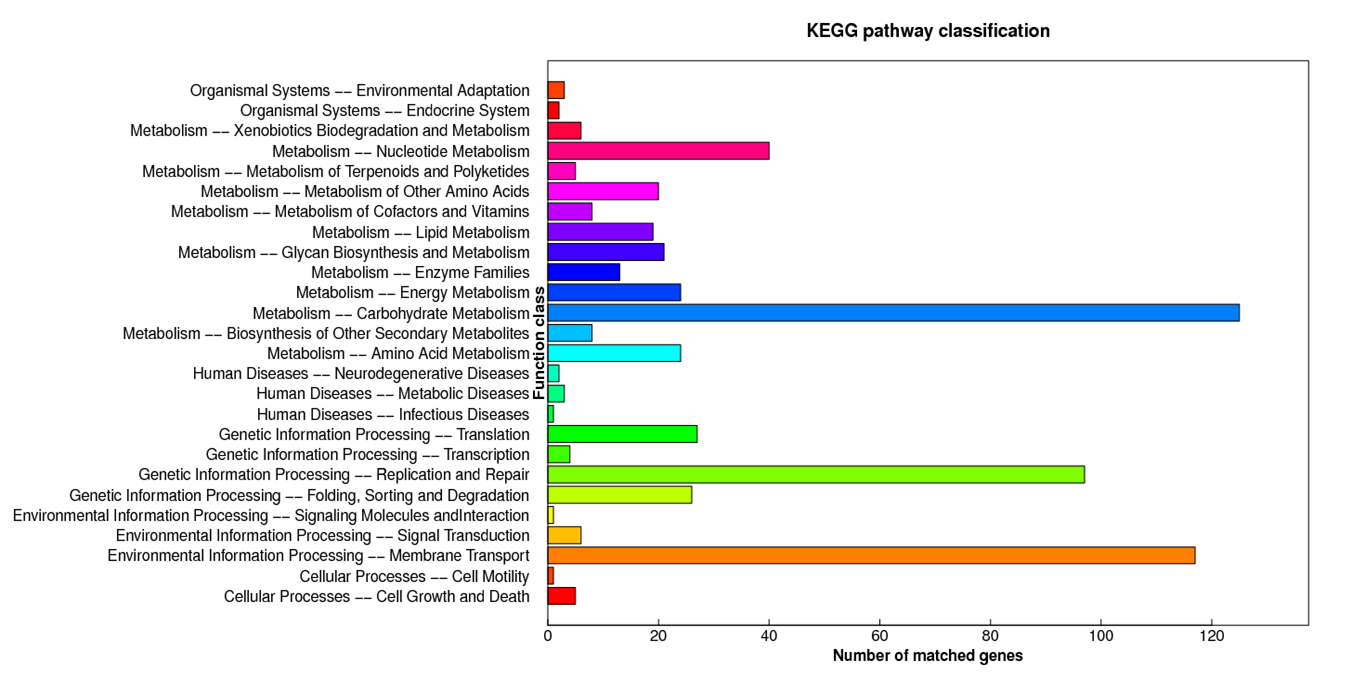

Supplement: Additional file 1: Tables S1, S2, S3, S4 — Shows the repeat sequences statistics, SNP, Indels between LCT-EF258 and LCT-EF90, and annotation of InDels respectively. Supplementary figure represent function annotation in GO, GOG and KEGG database. [file 1471-2180-13-304-S1.doc]
